# Multifocal Electroretinography in the Presence of Temporal and Spatial Correlations and Eye Movements

**DOI:** 10.3390/vision1010003

**Published:** 2016-05-09

**Authors:** Alan B. Saul, Amber E. Still

**Affiliations:** Department of Ophthalmology, and James and Jean Culver Vision Discovery Institute, Augusta University, Augusta, GA 30912, USA

**Keywords:** multifocal ERG, eye movements, decorrelation, retinal function, scotoma, kernel analysis

## Abstract

Releasing patients from the fixation task, and permitting them to view natural stimuli such as movies, would provide increased comfort, and potentially additional signs of retinal function, when recording multifocal electroretinograms (mfERGs). Techniques must be developed to handle the difficulties that arise from these alternative stimulation strategies. Multifocal stimuli were presented to volunteer human subjects with and without fixation. Retinocentric analyses were performed to deal with shifts of the stimulus across the retina in the presence of eye movements. Artificial scotomas that moved with the eyes to simulate local retinal defects were presented to assess whether such defects might be detectable in the presence of eye movements. Temporal and spatial correlations in the stimulus can be discounted, permitting retinal kernels to be measured in response to natural stimuli. Responses to temporally natural stimuli tend to have slightly stronger amplitudes because of the presence of low temporal frequencies in these stimuli. The effects of eye movement artifacts can be reduced, permitting similar kernels to be obtained in the absence and presence of eye movements. Convergence to stable kernels took slightly longer in the presence of temporal correlations or eye movements. Artificial scotomas can be localized with these methods. It may be possible to perform multifocal ERG recordings in the clinic using more flexible, natural techniques. However, work is needed to achieve results comparable to those routinely obtained with conventional methods.

## 1. Introduction

Temporally and spatially correlated stimuli can be used for multifocal electroretinography (mfERG), in contrast to the conventional use of independent stimulus elements modulated with temporally white luminance sequences [1,2,3]. These correlations must be discounted in order to properly assign credit for responses to the stimulus elements that caused those responses [4,5,6,7,8,9].

Eye movements can also be allowed, as opposed to requiring steady fixation [3,10]. Two problems emerge because of the presence of eye movements during recording. Each saccade evokes an artifactual signal that interferes with recording the desired retinal activity. These artifacts need to be discounted [3,11]. In addition, movement of the eyes across the stimulus moves the stimulus across the retina, so analyses can no longer be performed in stimulus coordinates. Instead, analyses must take place in retinotopic coordinates, and the stimulus modulations over time must be computed based on known stimulus and eye position records.

We show briefly that temporally natural stimuli can be used, and primarily address questions involving spatial issues. Can accurate mfERGs be obtained in the presence of eye movements? Can results be obtained in reasonable sampling times?

This work might lead to additional capabilities of mfERG, especially in testing younger and older patients more effectively. Eventually, it may be possible to evaluate geographic retinal function by letting patients watch natural movies. The general goals are to make mfERG more patient-friendly, more natural, more quantitative, and easier for the clinician to interpret.

## 2. Results

### 2.1. Temporal Correlations

ERGs were obtained using stimuli that contain temporal correlations. Subjects were tested with full-screen natural and Gaussian white noise. These stimuli had matched contrasts (RMS contrast = 0.29) and mean luminance (100 cd/m^2^). Each frame of the Gaussian white noise was chosen independently from a Gaussian distribution, whereas for the natural noise, the luminance of each frame depended on previous frames (see Section 4.3). The amplitude spectrum of the natural noise was relatively constant as a function of log temporal frequency, with more power at low frequencies and less at high frequencies compared to the white noise. The power was similar around 10 Hz.

**Figure 1:**
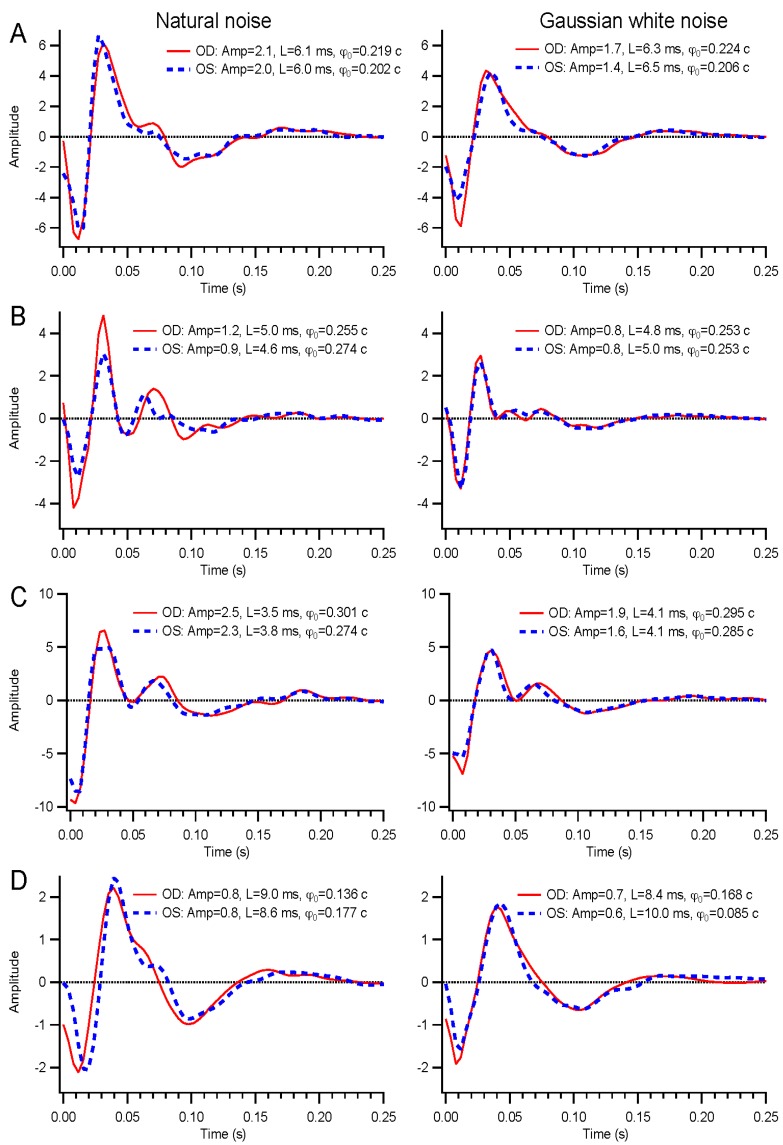
Basics of kernels. Examples of kernels from the right (solid red) and left (dashed blue) eyes of four subjects are illustrated, for natural noise on the left and Gaussian white noise on the right. Measured parameters are given as amplitudes (arbitrary units), latencies, and absolute phase. (**A)**, 76-year old female; (**B**), 55-year old female; (**C**), 49-year old male; and (**D**) 27-year old female.

Examples of kernels are shown in Figure 1. The solid red and dashed blue traces represent the right and left eyes, respectively. The initial deflection is negative, related to the a-wave in flash ERG responses. This is followed by a positive phase. The positive deflection often has two modes, a feature that varies considerably. Amplitude, latency, and absolute phase values are listed in the figure legend for each kernel (see Section 4.5 for definitions of these parameters). The earliest portion of the kernels, prior to the latencies of 5–10 ms, can be ignored.

Results for Gaussian white and natural noise are similar. The main differences in kernels lie in the early and late phases. In the frequency domain, these differences are much clearer, as amplitudes at low frequencies are consistently weaker for white noise stimuli. This is because white stimuli are impoverished at low frequencies. Even though the analysis normalizes for the stimulus statistics, white stimuli do not evoke sufficient responses at low frequencies to be observable in most instances.

#### 2.1.1. Population Results for Temporal Correlations

**Figure 2:**
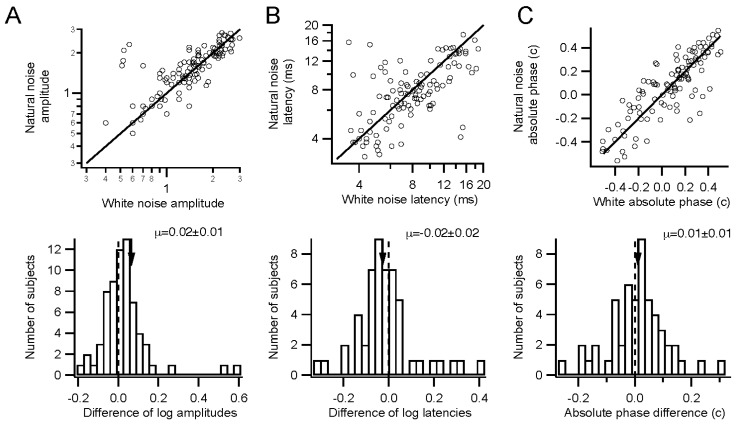
Discounting temporal correlations. (**A**) Amplitudes (arbitrary units) measured across a population of 67 subjects for natural (vertical axis) and white noise stimulation are plotted (**upper graph**). The ratio between the natural and white noise amplitudes is displayed as a histogram (**lower graph**) after averaging across the two eyes for each subject. Log mean ratio of amplitudes (arrow) is 0.02 ± 0.01 (*p* = 0.1, paired *t*-test), corresponding to 6% higher amplitudes for natural noise; (**B**) Latencies are plotted as in A, with log scaling. The log mean ratio of latencies is −0.02 ± 0.02 (*p* = 0.26). This corresponds to a mean ratio of latencies of 0.96; (**C**) Absolute phase values are plotted as in A. The mean difference in absolute phase is 0.01 ± 0.01 c (*p* = 0.36). Absolute phase is a cyclic variable, so −0.5 c is the same as +0.5 c.

Comparisons such as those in Figure 1 were made between responses to stimuli with differing temporal statistics. Across the population, amplitudes were marginally larger for natural than for Gaussian white stimuli (Figure 2A). Timing only differed slightly, if at all. Latencies were similar and absolute phase values were slightly retarded for natural stimuli compared to white stimuli. Latencies had little variance, falling between 4 and 10 ms generally (Figure 2B). Absolute phase values were 0.01 c later on average (Figure 2C). The absolute phase values had substantial variance across the population, with a range of more than a quarter cycle. The correlation in Figure 2C is 0.7, indicating that kernels were similar for the two stimuli. Latency and absolute phase tend to be anticorrelated, with the linear regression of phase *vs.* frequency pivoting around a frequency of about 40 Hz. In summary, temporally natural stimuli can evoke kernels that tend to have slightly larger amplitudes and slightly later absolute phase values, but are otherwise similar to those obtained with white stimuli.

#### 2.1.2. Convergence

**Figure 3:**
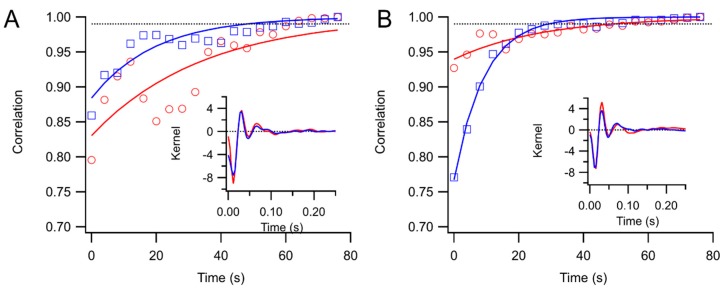
Examples of kernel convergence. Correlations of running kernel estimates with the final kernel are shown against testing time. The curves are exponential fits. The dotted line is at 0.99. The final kernels are shown in the insets. Traces with red circles are right eye and with blue squares left eye. (**A**) Natural noise; (**B**) Gaussian white noise.

Convergence to asymptotic kernels was similar for white and natural stimuli. Figure 3 illustrates how kernels approached the form of the final kernel over a sequence of trials presented to a subject. In many cases, after just one or two trials, the kernel captured its final form. In other cases, usually because of the presence of artifacts, convergence was slower (right eye in Figure 3A). Averaging over 66 of the subjects, it took 74 ± 2 s for kernels to reach the 99% correlation point with the final kernel for natural stimulation, and 66 ± 2 s with white stimulation (*p* < 0.01). The mean log ratio of the convergence times for natural and white full-screen stimuli was 0.07 ± 0.04 (*N* = 66, not significant). Convergence was thus slower for natural stimuli, but the difference amounted to an increase of less than 10% of the testing time.

### 2.2. Multifocal Tests

Multifocal ERGs were obtained using stimuli that contain spatial correlations, and in the presence of eye movements. In order to analyze responses to these stimuli, novel methods were needed. The major paradigm shift is to no longer rely on the concept of stimulus elements, since these are not tied directly to the retina, and are not independent. Instead, we consider an arbitrary set of positions across the retina. Note that results are shown in visual field coordinates, inverting the retinal coordinates.

#### 2.2.1. Retinotopic Analysis

**Figure 4:**
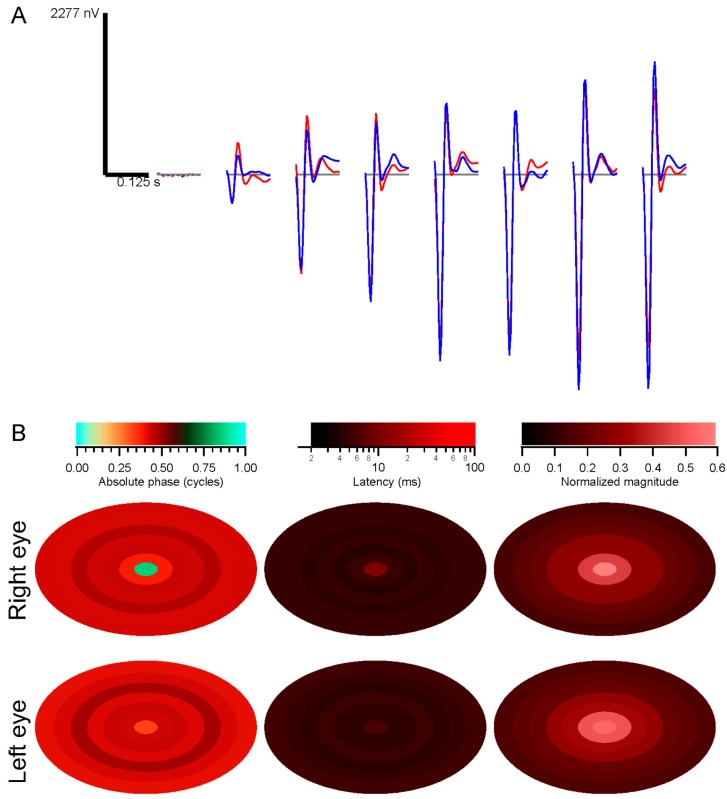
Conventional analysis: (**A**) subject was tested with seven concentric rings. Kernels from each ring are shown, moving from the central ring on the left to the most peripheral ring on the right. Kernels at the far left are controls. Red traces are from the right eye, blue from the left eye; (**B**) Summary of parameters measured from the kernels. Absolute phase, latency, and normalized magnitude are illustrated as pseudocolor plots against the stimulus spatial configuration. Magnitudes were normalized by stimulus area.

As explained briefly in Section 4.6, the conventional analysis method, based on computing kernels for each stimulus element, can not be applied in the presence of eye movements. Kernels were instead computed over a grid of retinal positions. For comparison, Figure 4 presents the conventional method of analyzing data in terms of stimulus elements. The stimulus in this case was a set of seven concentric rings, so that spatially separated positions on the retina (e.g., 15° inferior and superior) saw highly correlated luminance modulations. The subject fixated, so that the analysis provides information about the average responses as a function of eccentricity. The strongest kernels arise from the more peripheral rings, because they have larger areas. To measure response density, the raw amplitudes must be normalized by stimulus area (Figure 4B). In the presence of eye movements, retinal locations would be less correlated, but this stimulus-based analysis would not enable observation of this decorrelation.

**Figure 5:**
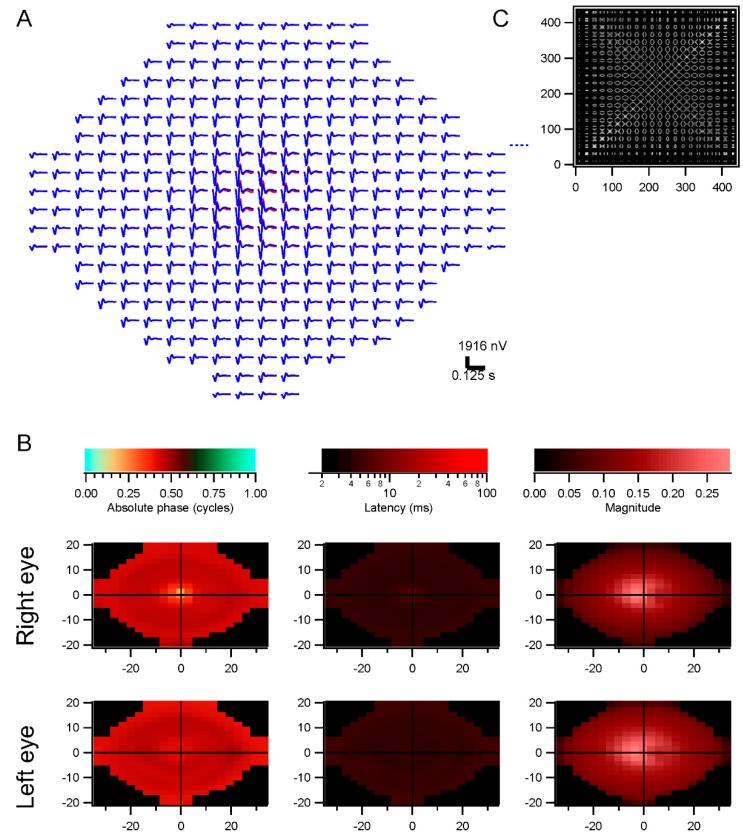
Retinotopic analysis. The data shown in Figure 4 were analyzed across 441 retinal locations. (**A**) Trace array showing the kernels derived for each retinal location. Red traces are from the right eye, blue from the left eye; (**B**) Parameter plots as in Figure 4B but across retinal locations; (**C**) Mean correlations across the 441 locations. Some locations were rejected because of low contrasts.

We therefore performed the analyses in retinocentric coordinates. A rectangular grid of points across the central visual field was chosen, and, for each grid element, the stimulus luminance profile over time was derived from the records of the stimulus and eye position. Movie S1 (Supplementary Materials) illustrates this retinocentric examination of the stimulus, showing the analysis grid that is purely virtual and was not present or visible during testing, along with a time indicator added here only for clarity. The average luminance in each grid element on each frame was computed. This set of retina-based stimuli was correlated with the response from each eye, rather than using the raw stimulus elements. Figure 5 illustrates the results from the same run as in Figure 4. The kernels in the trace array (Figure 5A) are similar to those in Figure 4A, although the amplitudes now have their natural scaling, rather than needing to be normalized by stimulus area. Positions to the right of fixation appeared to have weaker responses for some reason, perhaps because of the interaction of the subject’s fixation behavior with the stimulus configuration; this was not apparent with conventional analyses.

The spatial correlations in the stimulus used for this example (Figure 5C) were discounted using a spatial decorrelation technique described in Section 4.6. Because responses were similar across the retina, it is difficult to discern from this example whether that spatial decorrelation had its intended effect, although kernels on the right and left show some differences. With normal subjects, it can be difficult to overcome this absence of functional variability across the retina.

**Figure 6:**
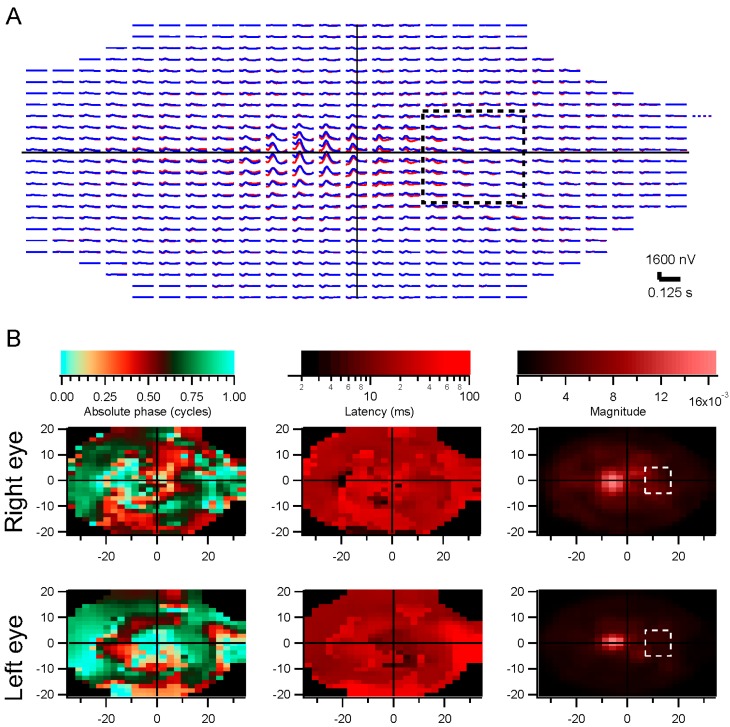
Artificial scotoma. (**A**) 3 × 8 dartboard stimulus was displayed, with a 10° × 10° region on the right side of the screen covered with an opaque patch. The subject fixated over 155 4 s trials. The trace array is shown in A, with the dotted square indicating the approximate position of the scotoma, which actually moved across the retina with eye movements. Red traces are from the right eye, blue from the left eye; (**B**) Parameter plots, showing the effects of the scotoma.

As a simple way to get around this, we made an artificial scotoma by simply covering a region of the display monitor. Figure 6 displays results from an experiment. The scotoma covered a 10° × 10° region of the screen. Because of eye movements (small fixational movements and occasional breaks in fixation), the scotoma was variable on the retina, so responses appear within the region, just as they appear over the blind spot. However, amplitudes are greatly reduced over much of the region, even though stimulus correlations with visible parts of the screen were strong. The peak amplitudes were shifted away from the scotoma (Figure 6B). We show below that scotomas fixed on the retina also result in reduced responses. First, we must consider the effects of eye movements.

#### 2.2.2. Discounting Eye Movements

**Figure 7:**
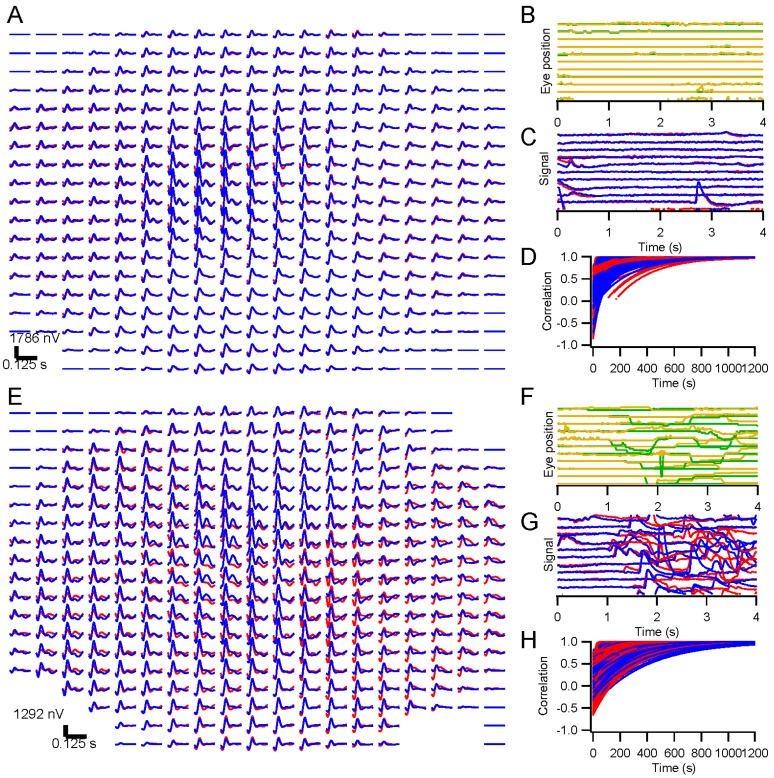
Release from fixation. In 300 interleaved 4-s trials, a subject was asked to fixate or allowed to move her eyes, with short lines of text to read in order to evoke saccades. The stimulus consisted of binary white noise modulations of a dartboard pattern across a 69° × 41° field. Trace arrays are shown in (**A**) (**fixation**) and (**E**) (**non-fixation**). Examples of 10 trials are shown in (**B**), (**C**), (**F**), and (**G**) for eye position records (green is horizontal and orange is vertical) and raw electrode signals (red is from right eye and blue from left eye). Convergence to the final kernels is illustrated in (**D**) and (**H**), showing the correlation of each kernel after each trial with the kernel after the final trial. There were 161 fixation trials and 139 non-fixation trials, so convergence times were about half of what is indicated in (**D**) and (**H**).

Subjects were tested with interleaved trials on which they were asked to fixate or could look around the screen. The difference between these conditions is not absolute, but the number of eye movements was far greater in the non-fixation than in the fixation condition. Kernels were derived for these two conditions and compared. Figure 7A,E illustrate the trace arrays for such an experiment. The kernels from the fixation trials are illustrated in A, and from the non-fixation trials in E. Insufficient data were available at a few of the 361 positions because stimulus contrast was too low on most trials. Panels B and F show the eye position records from representative subsets of 10 trials. On the fixation trials, eye position was fairly constant, but on non-fixation trials the subject was permitted to read a line of text on the screen, making a series of fixations. Eye movements lead to artifacts in the electrode signals, as can be seen in panels C and G. The recordings in G are clearly filled with artifacts. Despite these disturbances, the kernels in E are similar to those in A. For example, a late positive response occurs through the left eye at some central positions in this subject under both conditions.

##### Convergence with Eye Movements

One would expect that, given enough recording time, kernels can be extracted in the presence of artifacts, since portions of the records are free of artifacts. We compared the convergence time between the fixation and non-fixation conditions. Figure 7D,H plot the correlations between each kernel and its final form across recording time. Convergence times were significantly longer for the non-fixation condition (340 ± 4 s *vs.* 255 ± 5 s for the fixation condition). Kernels can thus be obtained in longer but still reasonable recording durations even in the presence of eye movements.

##### Similarity of Kernels with and without Eye Movements

**Figure 8:**
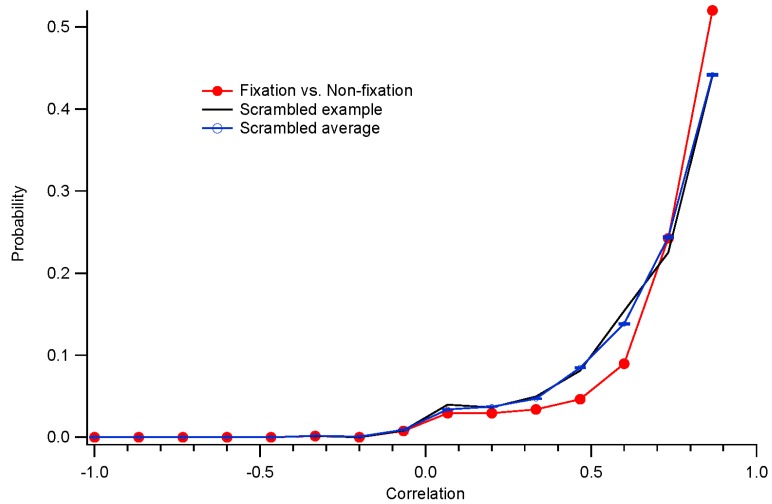
Histograms of correlations of kernels across fixation and non-fixation conditions. The red curve with solid circles shows the distribution of correlations between corresponding locations. The black trace with no symbols is an example of the correlations between scrambled positions. The blue curve with error bars shows the average distribution between scrambled positions as a control comparison.

The convergence time analyses do not address the validity of the final kernels. To do that, we can compare the estimated kernels obtained in the different conditions. Taking advantage of the fact that the underlying kernels vary slightly across the retina, we measured the correlations between estimated kernels at the same positions, and compared those values to the correlations between estimated kernels at scrambled positions. This controls for the similar forms of all kernels: the scrambled (control) correlations are strong because of this similarity. The results for the run illustrated in Figure 7 are shown in Figure 8. The unscrambled correlations reach levels well above those expected from the control comparisons. This pattern was observed in 10 of 12 subjects. That is, non-fixation kernels were similar to those found with fixation.

Other measures of the similarity between results with or without fixation come from comparing parameter values. Magnitudes were well correlated (*r*^2^ = 0.57 when comparing log magnitudes). Absolute phase was also well correlated (*r*^2^ = 0.85) but variability was much higher in the non-fixation condition. Latencies were poorly correlated (*r*^2^ = 0.06) as they have limited variance. These parameters thus appear to be independent of the fixation task.

##### Artificial Scotomas Fixed to the Retina

The ability of these methods to detect localized defects in retinal function will ultimately depend on clinical trials. Using normal subjects, we extended the artificial scotoma experiments described in Section 2.2.1 to retinally-based scotomas. A star-shaped portion of the screen was drawn over in gray, and this region moved with the eyes in order to simulate a retinal defect. Because eye position had to be fed back in real-time to shift the scotoma appropriately on the screen, the ability to draw frames was slowed down further, and only simple stimuli could be shown at >60 Hz.

**Figure 9:**
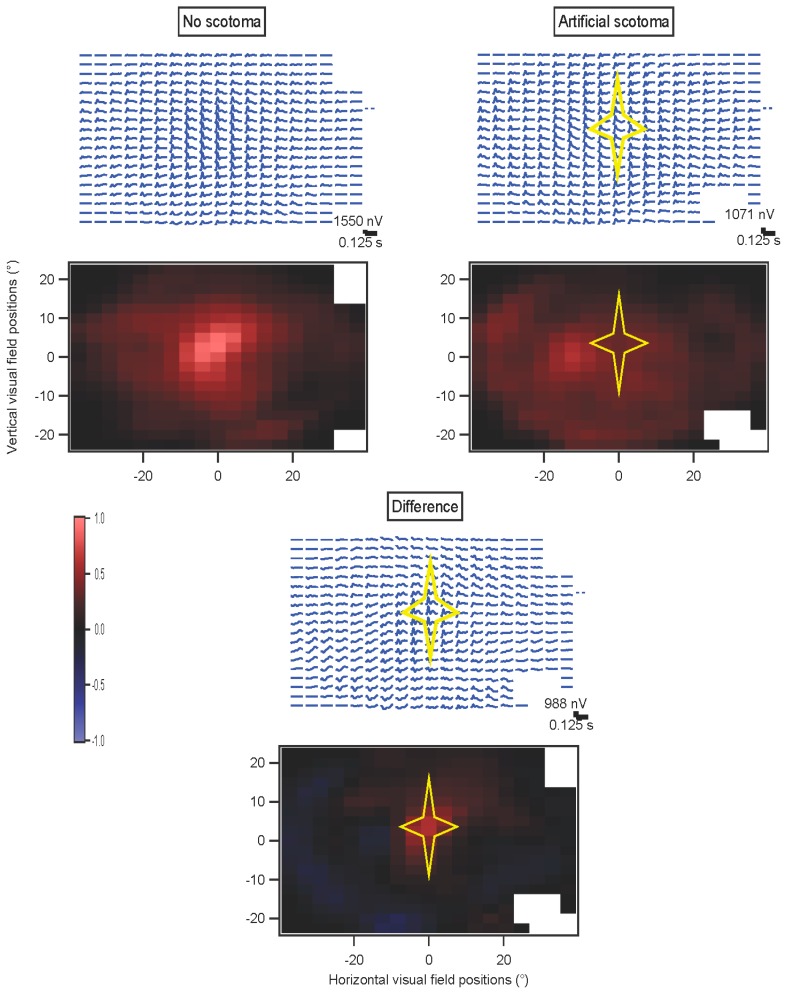
Artificial retinal scotoma. An example from the left eye of a subject is shown as visual field maps. The kernel arrays and the kernel values at 27 ms are shown for trials without and with a scotoma. At the bottom, the differences (**no scotoma minus scotoma**) are illustrated. The position of the star-shaped scotoma is indicated.

Figure 9 illustrates the results of such an experiment. The stimulus was a dartboard pattern with 12 elements. The subject was released from the fixation task for all of these trials, and on half of the randomly interleaved trials an artificial scotoma was present and moved with the eyes. Movie S2 (Supplementary Materials) illustrates the sort of stimulus that was on the screen during these experiments. The scotoma moves against the background stimulus pattern because of the subject’s eye movements, but is fixed on the retina within our limits of resolution. Because the stimulus had so few elements, correlations were high across the retina even in the presence of eye movements, so the scotoma is not as well-localized as might be possible with a more complex stimulus. There are also small edge artifacts from low contrasts along the outside border of the stimulus that were not sufficiently rejected. The kernel value at 27 ms, at or near the peak, was taken as an amplitude measure here in order to obtain both positive and negative values for the differences, in principle. The normal peak at the fovea was not seen in the presence of the scotoma. The difference map shows that the scotoma can be detected, and localized within about 5° at least centrally. Away from the scotoma, only small differences are observed.

**Figure 10:**
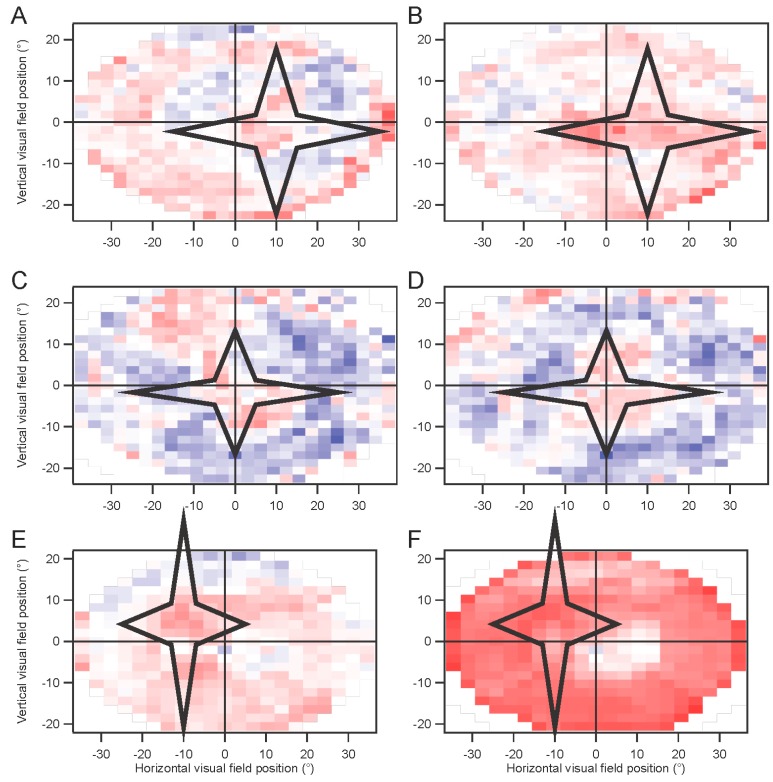
Artificial retinal scotoma. Examples from both eyes in three subjects are shown as visual field maps of the index based on amplitudes without and with a scotoma. Left eyes are shown on the left (**A**,**C**,**E**). Red color indicates positive indices, meaning smaller responses in the presence of a scotoma. P values for the difference between indices of positions inside *vs.* outside the scotoma are: 0.35 (**A**); 6 × 10^−9^ (**B**); 4 × 10^−6^ (**C**); 3 × 10^−16^ (**D**); 8 × 10^−7^ (**E**); 0.014 (**F**), via *t*-tests.

Strong statistical differences were seen in most cases between positions inside and outside the scotoma. We used larger scotomas, placed at various positions on the retina, to determine whether the difference between the scotoma and non-scotoma trials might be significant. Values of an index (difference divided by sum) based on the kernel amplitudes, rather than the kernel value at a single point as in Figure 9, for the non-scotoma and scotoma trials, were compared for points outside *vs.* inside the scotoma, by *t*-test. Over six eyes from three subjects, significantly smaller responses were seen inside the scotoma for five eyes. Figure 10 illustrates the results of these experiments. The stimulus was a dartboard pattern with eight elements in A–D, and with 12 elements in E, F. For real-world use, patient data would be compared to normative data, as is the standard practice in most cases of clinical visual electrophysiology. The results shown in Figure 10, in contrast to Figure 9, suggest that detection of a large peripheral scotoma with high spatial resolution requires a stimulus configuration with sufficiently high spatial frequency content.

## 3. Discussion

### 3.1. Temporal Statistics and Contrast

Retinal function can be evaluated under somewhat more natural conditions than are standard. Stimuli can have natural temporal statistics, with lower contrast. Sampling times may be relatively long, in order to overcome low stimulus contrasts. Temporally natural stimuli did not require much longer sampling times than white stimuli, on the other hand.

More natural stimuli should let the clinician observe retinal function in a more relevant context. A considerable literature suggests that slowing down the conventional 75 Hz temporally white stimulus permits observation of certain response components more clearly [12,13,14]. Work remains to be done to clearly show that these components are clinically useful, however [15]. Low contrast stimuli have been used in several studies [16,17,18], though data are limited. Mydriasis is typically the most bothersome part of the experience for the patient, and that may be dispensable as well [19,20].

### 3.2. Release from Fixation

We examined whether patients might be released from the standard fixation task during multifocal electroretinography. The evidence suggests that multifocal ERGs can be obtained in the presence of eye movements. Relaxing the requirements for fixation should be advantageous for many patients who have difficulty holding still and staring straight ahead. These advantages come with disadvantages. Many of these involve the lengthy computations needed. Analyses cannot be performed in times at all comparable to those available in the conventional system [21]. Sampling time is also longer.

#### 3.2.1. Analysis Time

Although kernel computations are far more complex, they can be completed, even with relatively slow hardware, in reasonable amounts of time for most clinical purposes. In addition, by using faster and especially multiple processors, analysis time should decrease dramatically. These are highly parallel problems that can be usefully treated with graphical processing units that have become relatively inexpensive.

#### 3.2.2. Recording Time

Sampling times may remain relatively long, in order to overcome low stimulus contrasts and additional artifacts from eye movements. However, this is partly compensated by improved comfort for the patient. Watching a movie for tens of minutes may be a more pleasant experience for most patients than a few minutes of fixating on flashing patterns. We are gathering subjective data on how subjects feel about their testing experience.

#### 3.2.3. Eye Movement Artifacts

Remarkably, the artifacts generated by eye movements do not drastically interfere with measurements of retinal responses. This is partly due to the fact that saccade frequency is low enough and saccade amplitudes are often small enough that a substantial portion of the recording time occurs while the eyes are only moving slowly. The artifacts generated by small saccades have durations shorter than the typical intersaccadic interval, so that retinal responses can be seen between saccades without artifacts present. Combining this fact with analysis techniques that remove artifacts from consideration permits extraction of accurate kernels [22]. Brute force methods of discounting artifacts can be problematic [11]. Our methods rely largely on median filtering of amplitudes, and especially on the reliance on phase that is not sensitive to artifacts.

#### 3.2.4. Decorrelation

Eye movements help to decorrelate the stimulus across space and time from the point of view of any retinal location [23,24,25]. The spatial decorrelation performed here is facilitated greatly by this fact. During fixation conditions, the spatial correlation matrix is highly singular, but becomes less so during non-fixation conditions.

### 3.3. Advantages and Disadvantages

Among the flexible aspects of these methods, we can arbitrarily choose both the stimulus configuration and the analysis grid. For example, these can be customized to match features in a visual field, OCT, or fundus photo. The stimulus can be optimized to reveal pathologies at particular locations. The flexible methodology provides additional choices that can be seen as complicating clinical decision-making, but in many cases simplifies testing and makes it more efficient. For example, a common protocol is used for monitoring patients who have a long history of hydroxychloroquine use. Testing is conventionally done with a set of hexagonal stimulus elements that are then averaged across rings, to detect the typical pattern of bullseye maculopathy. By instead making the stimulus a small set of rings, more power is assigned to the stimulus, and the analysis is clearly simplified.

These methods have been used successfully with dozens of patients. One example of their utility comes from testing a patient with macular degeneration, who had some nystagmus and many fixational saccades during testing. The stimulus-based analysis did not yield usable results except in the periphery, but the retinocentric method produced clean trace arrays, showing a strong peak a few degrees above the nominal foveal center.

Testing can be made more patient-friendly. A high percentage of patients are photophobic. Reducing temporal contrast is one way of making the experience more comfortable. Most people find it difficult to fixate for long periods of time, so releasing them from the fixation task makes it possible to record under more relaxing conditions for lengthier sessions. Giving patients something to watch that captures their attention and interest should be an additional bonus. Children could watch cartoons, for example, that would help to distract them from the sometimes intimidating environment of the clinic. Head-free gaze tracking is available that enables relatively free viewing.

Retinocentric analyses provide a novel means to focus on the ultimate goal, locations on the retina rather than on the screen. They make it possible to explicitly permit eye movements while recording. In addition, fixational eye movements and breaks in fixation can be easily handled by measuring eye position and regenerating the stimulus in retinal coordinates.

The ISCEV standards for mfERG provide crucial guidelines for clinics to provide high quality, consistent reports [3]. The modifications we describe here deviate significantly from the conventional techniques, unfortunately, and considerable work will need to be done to bring these methods up to those standards. In particular, the efficiency and reliability of the m-sequence technique must be approached, with the ability to obtain robust kernels in brief testing times.

### 3.4. Spatial Decorrelation

The conventional system empirically scales the stimulus elements from center to periphery in order to achieve approximately equal signal-to-noise values for the kernels across the retina. Response density is then computed by dividing each kernel’s raw magnitude by the area of the corresponding stimulus element. That process shows that response density peaks sharply at the fovea, correlated with cone density. However, this normalization can produce a spurious central peak when kernels are noisy. When performing a retinotopic analysis, the division by stimulus element area is replaced by normalizing by the stimulus correlations, such as via subtracting contributions from other retinal locations through the iterative decorrelation process. When using scaled stimuli, peripheral locations have stronger correlations with other locations than do central locations, so that their raw kernels are reduced.

The central kernels are typically not as strong as expected with our methods. This may be due to imperfect decorrelation, since the large stimulus elements we use do not isolate the fovea, so central responses are contaminated by those outside the fovea.

The phases of the stimulus correlations could be important, for example in situations where eye movements create systematic movements across the retina, or if the stimuli themselves move systematically as in natural movies. The correlation phase captures how stimuli move from one retinal position to another. Those movements need to be discounted, so that, for instance, responses from one retinal region are not attributed as later responses from the part of the retina to which the stimulus evoking the responses moves.

How well the decorrelation works remains to be determined. The artificial scotoma experiments, along with clinical trials, will enable testing of these methods. In the presence of eye movements, stimuli are more decorrelated across a long span of testing time. That should further indicate how well the decorrelation, applied during fixation trials, matches the non-fixation results.

## 4. Material and Methods

### 4.1. Subjects

Full screen and/or multifocal ERGs were obtained from 68 healthy subjects. They were recruited by advertising and word-of-mouth. Geometric mean age was 28 years, range 14–81 years, 74% were women, 43% were African-American. Subjects provided written informed consent and assent after the procedures and potential consequences were explained in full. All procedures were approved by the Institutional Review Board of Georgia Regents/Augusta University Medical Center (611231-6, 1 June 2014), and complied with the Declaration of Helsinki.

### 4.2. Recording Preparation

Drops of Proparacaine HCl (0.5%), Tropicamide (1%), and Phenylephrine HCl (2.5%) were applied to each eye for anesthesia and mydriasis. Areas of skin where reference and ground electrodes would be placed were scrubbed with alcohol pads. Reference electrodes were clipped to the ear lobes, and a cup electrode was taped to the forehead as a ground. Skin electrodes were filled with conductive gel. DTL-Plus electrodes (Diagnosys LLC, Lowell, MA, USA) were carefully placed across each eye, avoiding lashes and situating the adhesive pads so that the fiber ran directly along each canthus. Subjects reported not feeling the electrodes in nearly every case, even after more than an hour.

A table with the stimulating monitor and a chin rest was positioned at a comfortable height, and the chin rest was adjusted to accommodate the subject’s head. A video camera with infrared illumination (Arrington Research 220, Scottsdale, AZ, USA) was focused on one eye. Settings were made to optimize capture of the pupil, and a calibration was performed with the Arrington software. Voltages scaled across the eye position range of the monitor were sent from the Arrington system to a digitizer (National Instruments NI-6323, Austin, TX, USA). Another calibration was performed using custom software in Igor Pro 6 (WaveMetrics, Lake Oswego, OR, USA) based on those voltage signals. Any time the experimenters suspected it might be needed, an additional calibration was performed. During recording, each trial was preceded by recording eye position until it stabilized as the subject fixated, and these records were used as slip corrections to compensate for small head position changes.

Electrode signals were led to a PsychLab (Cambridge, MA, USA) EEG8 amplifier. Typically, gain was 10,000×, and the amplifier filtered the raw signal between 1 and 200 Hz. No notch filter was used. The amplifier output was digitized by the same NI-6323 DAQ (National Instruments, Austin, TX, USA) simultaneously with the eye position signals.

### 4.3. Visual Stimulation

Subjects viewed stimuli on a Samsung (Ridgefield Park, NJ, USA) 2233RZ 120 Hz LCD monitor at a distance of 29 cm. The viewing area on the monitor subtended about 70° × 40°, and the maximum brightness was set to 200 cd/m^2^. The stimuli took advantage of the aspect ratio of the screen, extending horizontally about 175% of the vertical extension. This monitor has excellent timing [26]. The time when each frame was presented was stored for synchronization with the electrode signals.

Stimuli were drawn on the screen by code written in Igor Pro. A fixation target, consisting of a diagonal cross and a circle, was present either during the initial 500 ms, or throughout the 4 s trials. Each trial was preceded by early appearance of the fixation target and either a message on the screen or an audible tone alerting the subject to the onset of the trial, so that they would fixate, and slip correction data (see Section 4.2) could be measured. The mean luminance of the screen was maintained constant at 100 cd/m^2^. Subjects could request a break at any time during sequences of trials. Intertrial intervals were typically 4 s.

A wide range of temporal modulations could be applied to the stimuli. For this report, we describe results from binary white, Gaussian white, and natural noise modulations. Binary white stimuli had the luminance of each frame chosen from the minimum and maximum luminance levels based on a pseudorandom number. Gaussian white stimuli contain a continuous, normally-distributed set of luminance values chosen independently on each frame. Natural noise is also continuously distributed, but the luminance on each frame depends on the previous frame’s value, *L*_n_ = *f*(0.9*L*_n-1_ + 0.1γ), where γ is a normally-distributed random variable with zero mean and standard deviation of 0.37, and the function f is a sigmoid that enhances contrast and bounds the luminance values between −1 and 1, to be subsequently rescaled to the range 0 to 200 cd/m^2^. The temporal contrast of the binary noise was 1, but the continuously distributed stimuli each had contrasts of about 0.29.

### 4.4. Experimental Protocols

We present results in this report from ERG testing with either full-screen, dartboard, or hexagonal patterns. Eye position was monitored in all cases with the Arrington pupil tracker. For some experiments, subjects were instructed to fixate throughout the run. Other experiments were designed to examine the effects of releasing the subject from the fixation task. For direct comparison, randomly interleaved trials were presented under the fixation and non-fixation conditions. Subjects were instructed to maintain fixation when the fixation target was present, but to move their eyes on trials where the target disappeared after 500 ms. Short lines of text (excerpts from Laozi, Lewis Carroll, Langston Hughes, Sean Singer, Maya Angelou, and Tagore) positioned randomly on the screen were provided on non-fixation trials so that subjects had something to look at and move their eyes across.

In some experiments, a star-shaped portion of the screen was drawn in gray over the multifocal stimulus. This artificial scotoma was shifted with the eyes, to maintain its retinal position (Movie S2, Supplementary Materials). These experiments were performed using four randomly interleaved conditions: fixation trials with no scotoma; fixation trials with scotoma; non-fixation trials without scotoma; and non-fixation trials with scotoma. Results for each of the four conditions were extracted separately. The kernels for the scotoma conditions were then subtracted from the kernels for the non-scotoma conditions. A comparison was computed for the non-fixation conditions, where an index (difference divided by sum) comparing the non-scotoma and scotoma amplitudes was plotted to provide a statistical measure of the effects of the scotoma.

Because of hardware and software limitations, presentation rates above 60 Hz could only be achieved for multifocal stimuli with a limited number of elements. This was especially true when artificial scotoma position was updated and redrawn in real time, which slowed processing.

Horizontal positions were corrected for the offsets of the two eyes. However, figures are shown with matching locations of the kernels, for clarity. Amplitudes have been scaled with these corrections.

For the experiments using natural temporal stimuli, only full-screen stimuli were used, to focus on the temporal issues, and inversely for the multifocal stimuli, only binary white temporal modulations were used, in order to focus on the spatial issues.

### 4.5. Wavelet Correlations

Responses were correlated with stimuli via the wavelet correlation method [9]. The goal of the analysis is to estimate the kernels that relate stimuli to responses. The first-order kernel is simply the response divided by the stimulus, in the frequency domain. Transforming the frequency domain kernel to the time domain yields the more familiar version of the kernel, also known as the impulse response function.

Each 4 s record of the stimulus luminance and electrode voltage was represented in the time-temporal frequency domain via a complex continuous Morlet wavelet transform. This representation provided a detailed view of amplitude and phase of the signals at 2048 time samples over 37 temporal frequencies ranging from 0.25 to 150 Hz.

The stimulus wavelet was filtered to remove low contrasts (below 1% of the average contrast across all frequencies and times). The response wavelet was then divided by the stimulus wavelet, to provide an estimate of the kernel at each time sample. A key step was then imposed: at each frequency, the amplitude of the kernel estimate was median filtered across time to remove artifacts. The median filter looked over ±1 s around each time point, and if the amplitude deviated from the median over those 2 s by more than the standard deviation of the amplitude over the whole 4 s trial, the amplitude was instead set to the median. This median filtering was iterated until its effect was less than 5% of the maximum amplitude. Artifacts, including those evoked by eye movements, were greatly discounted by this procedure. Note that this median filtering was only applied to the amplitudes. Crucially, phase was unaffected, other than reducing the effects of artifactual phase values. This is different from median filtering in the time domain.

The filtered amplitude was then recombined with the phase of the kernel estimate (kernel phase is response phase minus stimulus phase), and for each frequency the average over the time samples was computed in the complex plane. Only times within the “cone of influence” were considered, omitting times near the beginning and end of the trial for low frequencies. The cone of influence included points where the wavelet transform provided amplitudes to sinusoidal tests within two standard deviations of the maximum amplitude at that frequency.

The kernel estimate was then interpolated to a function of 513 frequencies ranging from 0 to 128 Hz, avoiding extrapolation. Kernel estimates were averaged across trials. The same process was applied to derive control kernels, using a stimulus rotated in time by a random amount between 1.5 and 2.5 s to disrupt real correlations.

Note that all of these calculations are performed in the frequency domain. Kernels were only transformed to the time domain for illustration purposes.

Kernel characteristics were quantified primarily with three parameters. Amplitude is taken as the root-mean-square value of the impulse response over the first 500 ms. This is equivalent to the total power in the frequency domain [27]. Timing was measured with a linear regression of phase *vs.* frequency [28]. The slope is the latency, and the intercept is called absolute phase, measured in cycles. Absolute phase corresponds to the shape of the kernel. Absolute phase of 0 means a sustained response, with a unimodal shape. Absolute phase values just less than 0 arise from kernels with an initial positive mode followed by a smaller negative mode. A small initial negative mode followed by a larger positive mode produces a slightly positive absolute phase: this is the most common shape of photopic ERG kernels (*i.e.*, a-wave followed by b-wave).

Kernels accumulated after each trial were stored. These were compared to the “final” kernel after the last trial by correlating the impulse response functions. The correlation coefficients approach 1 asymptotically by design. Two measures of convergence rate were obtained: the time constant for an exponential fit, and the first time at which the correlation coefficient exceeded 0.99 and did not later fall below that level.

### 4.6. Retinotopic Analyses and Spatial Decorrelation

For the multifocal experiments, stimuli were discretized in space across the retina by choosing a grid covering an area larger than the most strongly stimulated portion of the retina. For each stimulus frame, the screen stimulus was shifted based on the eye position record, and the mean luminance in each retinal grid element was taken as the luminance value for that frame. The sizes of the chosen grid elements were smaller than the sizes of the stimulus elements, with at least 4–5 grid elements for each stimulus element, typically; however, contrast tended to be lower for grid elements located along stimulus element borders at central gaze.

The grid-based retinotopic stimuli were correlated with the responses from each eye, via the wavelet correlation method (Subsection 4.5; [9]). The kernels estimated in this way contain information not only about retinal function at the corresponding grid points, however, but are influenced by retinal function at other locations where the stimulus was correlated with the stimulus at the home position. These correlations need to be removed in order to isolate the contribution to each kernel only from its own retinal location. Decorrelation was performed as follows.

We make the assumption that the global ERG signal (*r*) arises from a uniform linear combination of the local ERG signals:

(1) $$r=\sum_{i}k_{i}*s_{i}$$

where *k_i_* are the kernels and *s_i_* the stimuli across locations *i*, and * is the convolution operator. In the frequency domain this becomes

(2) $$R=\sum_{i}K_{i}S_{i}$$

where the upper case indicates Fourier transforms of *r*, *k*, and *s* (these are functions of time, so their Fourier transforms are functions of temporal frequency).

Correlating the response with the stimulus at a location *j*, which is equivalent to multiplying both sides of Equation (2) by $S_{j}^{*}/{\left|S_{j}\right|}^{2}$, gives

(3) $$RS_{j}^{*}/{\left|S_{j}\right|}^{2}=\sum_{i}K_{i}S_{i}S_{j}^{*}/{\left|S_{j}\right|}^{2}$$

where the * indicates complex conjugation. This can be written as

(4) $$K^{0}=KC$$

where $K^{0}$ (superscripts are used to index over iterations below, so this is for the 0-th iteration) is the vector of kernel estimates prior to decorrelation, *K* is the vector of decorrelated kernel estimates, and *C* is an asymmetric correlation matrix

(5) $$C_{ij}=S_{i}S_{j}^{*}/{\left|S_{j}\right|}^{2}$$

We can thus solve for the decorrelated kernels:

(6) $$K=K^{0}C^{-1}$$

We compute the kernel estimates first as $K^{0}$, while compiling the stimulus correlation matrix *C*. We could then invert the correlation matrix and compute the decorrelated kernels. In general, however, the correlation matrix is highly singular, and pseudoinverse methods we tried were not effective. We have used an iterative method to compute the solution for *K*. Note that Equation (4) can be rewritten in a form that has intuitive appeal, as

(7) $$K_{j}=K_{j}^{0}-\sum_{i\neq j}K_{i}C_{ij}$$

because $C_{jj}=1.$ One can see that the initial kernel estimate *K*^0^ needs to be corrected by subtracting the unwanted contributions of other retinal locations. We compute these kernel estimates iteratively, so that for the n-th iteration.

(8) $$K_{j}^{n}=K_{j}^{0}-\alpha^{n}\sum_{i\neq j}K_{i}^{n-1}C_{ij}$$

until they converge, $||K_{n}-K_{n-1}<\varepsilon ||$, as α*^n^* approaches 1 from below, for ε small. Convergence is not guaranteed. For example, a full-field stimulus would mean that all correlations were exactly 1, and obviously would not permit local kernels to be distinguished. The iterative computations tend to be unstable, and we implemented controls to reduce this instability. The detailed methods are available from the author.

These calculations are extremely slow, and for the purposes of most of the results in this report, we show simpler decorrelations. Instead of using Equation (6) with complex correlations, we divide *K*^0^ by the real-valued instantaneous averaged stimulus correlations. Ignoring a phase like this only has minor effects in the current context, but becomes a problem when the stimulus contains coherent motion, as in natural movies, in addition to the coherent motion induced by smooth eye movements that are uncommon here [29].

## 5. Conclusions

Flexible, more natural methods should provide improved insights into retinal function. Temporal correlations can be discounted successfully in ERG recordings. Spatial correlations can also be discounted, as well as artifacts created by eye movements. However, considerable efforts will be required to approach the efficiency and resolution of conventional techniques.

## Supplementary Materials

The following are available online at http://www.mdpi.com/2411-5150/1/1/3/s1, Movie S1: Retinotopic analyses. The 7-ring stimulus used for the results in Figure 4 is shown in retinal coordinates. The subject fixated on the circle and cross target, but fixational errors and eye movements shift the screen stimulus on the retina as shown. The blue grid was not present for the subject, but shows how the retinal space was subdivided to provide a retinocentric version of the stimulus. The timer in the upper left corner was not present either, and shows the time during this trial. Many such trials were presented, and eye movements differed on each trial; Movie S2, Artificial scotoma. This is a screen view of an example of the stimulus seen by the subject during the experiment that generated Figure 9. The star-shaped artificial scotoma was relatively fixed on the retina, and therefore moved around the screen with her eye movements. Thus, the dartboard stimulus on the screen moved on the retina, and different elements were obscured by the scotoma at different times. The counter indicating the time was not present during the experiment, and is shown to provide a sense of the rescaling of the 4 s trial.
